# Crystallization Behavior of Isotactic Polybutene Blended with Polyethylene

**DOI:** 10.3390/molecules27082448

**Published:** 2022-04-11

**Authors:** Jiajia Ping, Guiqiu Ma, Zhe Ma

**Affiliations:** Tianjin Key Laboratory of Composite and Functional Materials, School of Materials Science and Engineering, Tianjin University, Tianjin 300072, China; pingjiaj@163.com

**Keywords:** polybutene, blends, crystallization, polymorphism, phase transition

## Abstract

In this work, the melt crystallization behavior and the solid phase transition of isotactic polybutene (PB) were studied in the polybutene/high-density polyethylene (PB/PE) blends covering the whole composition range. For the dynamic cooling crystallization, PE exhibits almost the same crystallization temperature in all blends, whereas PB exhibits a distinct non-monotonic dependence on the composition ratio. Combining the ex situ X-ray diffraction and in situ Fourier transform infrared spectroscope, it was demonstrated that during cooling at 10 °C/min, the presence of at least 70 wt% PE can induce the formation of form I′ directly from the amorphous melt. The detailed relations of polymorphism with temperature were systematically investigated for the PB/PE blends. Different from the formation of the sole tetragonal phase with ≤50 wt% PE, the trigonal form I′ could crystallize directly from amorphous melt with ≥60 wt% PE, which can be further enhanced by elevating the temperature of isothermal crystallization. Interestingly, the critical lowest temperature of obtaining pure form I′ was 85 °C with 70 wt% PE and decreased to 80 °C as the PE fraction was increased to 80 wt%. On the other hand, the spontaneous phase transition from the kinetically favored form II into the thermodynamically stable form I was also explored with X-ray diffraction methods. It was found that at the room temperature, phase transition kinetics can be significantly accelerated by blending at least 70 wt% PE.

## 1. Introduction

Polymer blending provides a robust manufacture strategy to obtain new materials with varying structures and to achieve complementary performance advantages of distinct components [1,2]. The final properties of the blend material are not only dependent on the individual features but also associated with their mutual influences on each other. Especially when the blend is composed of two crystallizable polymers, the component that crystallizes first could cause a significant influence on the other counterpart. It has been well recognized that for the crystallizable components in the blend, the kinetics and polymorphism of crystallization can be changed by the presence of the second component [3,4,5,6,7,8]. For example, the pure poly(vinylidene fluoride) (PVDF) prefers to crystallize into the nonpolar monoclinic α-crystallites, whereas blending PVDF with a low fraction (10 wt%) of poly(ethylene terephthalate) generates the substantial electroactive orthorhombic β-crystallites with a high content of up to 80%, so as to obtain the significantly improved piezoelectric properties [9]. For the biodegradable poly(L-lactic acid) with α′ crystals at T_c_ < 110 °C, the additional introduction of poly(ethylene oxide) is even able to induce the formation of pure α crystal with excellent mechanical and barrier properties [10]. Obviously, the quantitative correlation between crystallization behavior and blend composition is crucial for the material design and performance of materials.

The isotactic polybutene (PB) is a typical polymorphic polymer with excellent mechanical properties [11]. PB can crystallizes into three crystalline modifications, including the trigonal phase (form I/I′), tetragonal phase (form II), and orthorhombic form III [12,13,14,15,16,17,18,19,20,21,22,23]. Among these crystal modifications, the trigonal phase with a 3/1 helix is the most stable from a thermodynamic point of view. However, the tetragonal phase with an 11/3 helical conformation has a significantly larger crystallization kinetic advantage, where the linear growth rate is two orders higher than that of the trigonal phase [24,25]. Therefore, at atmospheric pressure, PB very easily crystallizes from amorphous melt into the kinetically favored form II [14,18,25,26,27,28]. Due to poor thermodynamic stability, the generated form II tends to further transform into the most stable trigonal form I, and this process could spontaneously happen at room temperature [29,30,31,32]. Although the transformed trigonal form I has improved thermal stability, its slow transformation kinetics from form II often takes weeks, leading to an elongated processing period and the resulting unstable property [33,34,35,36].

During the past decades, there have been many investigations devoted to studying the effective approaches to control the crystallization polymorphism of PB. It was found that there are available physical and chemical methods to accelerate the phase transition from form II into form I, such as the two-step annealing protocol [29,37], pressured CO_2_ [38,39,40], mechanical stretching [41,42,43,44], and the copolymerization with special co-units [45,46,47,48]. In addition to accelerating II-I phase transition, the direct formation of the trigonal phase from the amorphous melt, referred to form I′ to distinguished from the above form I (transformed from form II), also provides an alternative pathway to tune the crystal modification [49,50]. For this purpose, physical blending has been proven to be the available method to vary the crystallization polymorphism of PB. So far, the blending of PB was mainly studied with isotactic polypropylene (iPP) [21,23,51,52,53,54,55,56]. Shieh et al. found that mixing with iPP could not only accelerate the II-I phase transformation rate of PB but also promote the direct formation of the trigonal form I′ through changing the blend composition [51]. In addition, utilizing crystallization conditions of PB/iPP blends also affects the polymorphism behavior of PB [21]. Zhong et al. applied the two-step treatment on cold crystallization to directly generate the trigonal form in PB/iPP blends with low iPP content down to 10 wt% [23]. Afterwards, they further studied the influence of molecular weight on crystallization and found that the low molecular weights of both PB and iPP were favorable for form I′ formation [55].

Meanwhile, polyethylene is also one class of the most widely used crystalline polyolefin materials and its common crystal modification is its orthogonal phase in most crystallization conditions, except in severe conditions such as high pressure. Recently, Mohammadi et al. studied the crystallization behavior of PB blended with 5–20 wt% low density polyethylene [57]. They found that, under processing conditions, PB in the nanofibers with an average diameter of around 200 nm exhibit the facilitated formation of the trigonal phase and, interestingly, the further addition of organoclay hinders the generation of the kinetically favored form II. In reality, the fundamental correlation of crystallization polymorphism with composition ratio and temperature is still far from a comprehensive understanding. Furthermore, the related solid phase transition from the initial form II into the more stable form I remains unknown. 

In this work, a series of polybutene/high-density polyethylene (PB/PE) blends covering the whole composite range were prepared by a solution mixing method. The temperature dependence of polymorphism and the phase transition rate of PB blended with PE were systematically studied by differential scanning calorimetry, Fourier transform infrared spectroscopy, and the X-ray diffraction method. The results show that the crystallization behavior of PB is strongly dependent on the composition of blend materials. During cooling, the crystallization temperature of PB shows a non-monotonous dependence on the blend composite. What is more interesting is that blending with ≥60 wt% PE can effectively promote the formation of PB form I′ from the amorphous melt, which can be facilitated by the elevation of isothermal temperature. The quantitative relation between crystal polymorphism and temperature was systematically investigated for various blends. Moreover, the results also show that the solid phase transition from form II into form I was significantly accelerated by the presence of ≥70 wt% PE.

## 2. Experimental Section

### 2.1. Materials

The isotactic polybutene (PB) was produced by LyondellBasell (PB0800M), with a weight-average molecular weight of 77 kDa and a polydispersity index of 3.0 [37]. The high-density polyethylene (HDPE) was purchased from DOW Chemical Company (with the grade name of DMDM-8904), of which the weight-average molecular weight was 83 kDa and the polydispersity index was 3.8 [58]. The PB/PE blends were prepared by the solution–precipitation method, in which the contents of PB covered the whole range from 0 to 100 wt%. First, the blends of PB and PE granules (5 g in total) were dissolved in xylene at 130 °C, which were stirred under a nitrogen atmosphere for 2 h to ensure that the polymers were mixed completely. Next, the solution was poured into the excessive methanol, and the precipitates were collected and dried under vacuum at 60 °C for 72 h. The pure PB and PE samples were also processed in the same way. The samples were compressed by hot pressing at 180 °C for 5 min into the film samples for the following experiments. 

### 2.2. Methods

The differential scanning calorimetry (DSC) experiments were carried out with DSC Q2000 (TA instrument, New Castle, DE, USA) under a nitrogen atmosphere. The instrument was calibrated with indium and the sample weight taken for each DSC experiment was approximately 5 mg. The sample was first annealed at 190 °C for 10 min to erase the thermal history. Then, the relaxed melt was cooled to 25 °C at a rate of 10 °C/min to perform the dynamic cooling experiments. On the other hand, isothermal experiments were also performed at different temperatures. For isothermal crystallization, the temperatures studied varied from 65 to 100 °C.

The in situ Fourier transform infrared spectroscope (FTIR) measurements were carried out at a resolution of 4 cm^−1^ with an FTIR spectrometer IRtracer100 (Shimadzu, Japan). The hot stage, equipped with ZnSe windows, was used for the in situ FTIR characterizations where the thermal protocol applied was the same as the above DSC measurements. 

The X-ray diffraction experiments were conducted by XRD Ultima IV (Rigaku, Japan) to identify the crystalline modifications. The scanning range was 5–25° and the scanning rate was 5°/min. 

## 3. Results and Discussion

### 3.1. Cooling Crystallization

**Figure 1:**
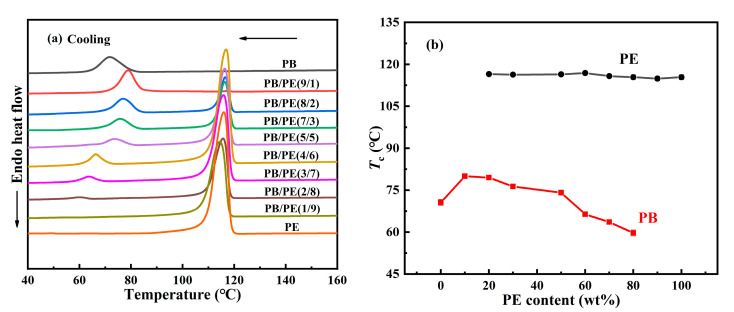
(**a**) DSC cooling curves of blends and pure polymers. (**b**) The cooling crystallization temperatures (*T*_c_) as a function of PE content.

Figure 1 shows the influence of the PE component on the cooling crystallization behavior of PB in the blend. It can be seen that, in all blends, PE crystallized first at a higher temperature than PB, which seems similar for different composition ratios. As the temperature was continuously cooled, PB started to crystallize at a lower temperature. Interestingly, the cooling crystallization temperature of PB shows a strong dependence on the blend composition. From Figure 1b, it was found that, with increasing the PE content, the crystallization temperature *T*_c_ of PB increases first and then decreases, different from the almost constant crystallization temperature *T*_c_ = 116 °C of PE. These results show that the introduction of 10 wt% PE can improve the crystallization temperature of PB from 70.6 °C in pure PB to 80.0 °C in PB/PE(9/1). As the PE content was increased further, the crystallization temperature of PB began to decrease and becomes even lower than that of pure PB with PE content ≥ 60 wt%. It should be explained that the component content of 10 wt% is too low to clearly identify its crystallization, the crystallization temperatures were not included for PE in PB/PE(9/1) or PB in PB/PE(1/9). When PE started to crystallize during cooling, the PB components remained in the molten state, and it seemed hard to affect the crystallization of PE. However, the completed PE crystallization introduced a complex confinement environment for the subsequent crystallization of PB, leading to a non-monotonous change in PB crystallization, as shown in Figure 1b.

**Figure 2:**
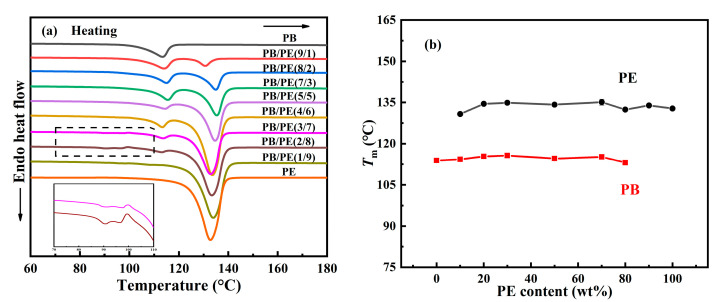
(**a**) DSC heating curves of blends and pure polymers. (**b**) The melting temperatures (*T*_m_) as a function of PE content.

Following this, the melting behaviors of the PB/PE blends were studied after the aforementioned cooling crystallization. From Figure 2a,b, it can be seen that PB first melts at a relatively lower temperature of approximately 113 °C and PE melts at an elevated temperature of approximately 133 °C. It is obvious that both of the PB and PE melting temperatures were independent from the components. For PE, it is expected to exhibit similar melting behaviors, regardless of composition, since its cooling crystallizations happened at the same temperature, as shown in Figure 1. In contrast, the constant melting temperature (*T*_m_), which originated from the various *T*_c_, suggests that the formed crystallites experience a distinct melt–recrystallization process to reach the similar lamellar thickness in the ultimate melting [59,60,61]. Interestingly, additional melting peaks were observed in the lower temperature range (85–98 °C) for blends with high PE contents, i.e., PB/PE(3/7) and PB/PE(2/8). This may be associated with the formation of the trigonal form I′ of PB that was generated directly from the amorphous melt, which will be discussed in the following section.

**Figure 3:**
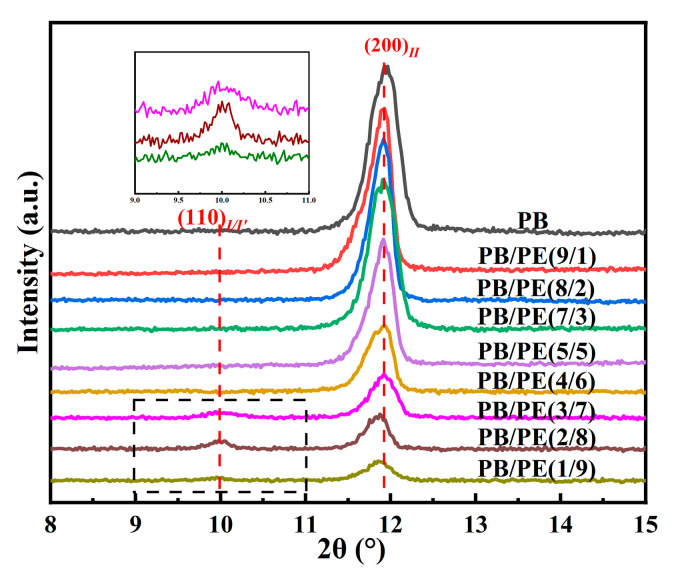
The X-ray diffraction (XRD) curves of PB and PB/PE blends obtained by cooling crystallization.

In order to disclose the crystalline modification of cooling crystallization, XRD experiments were performed on the samples prepared with the same thermal process as the above DSC experiments. As shown in Figure 3, a distinct diffraction peak was observed at 2θ = 11.9°, which corresponds to the (200)_II_ crystallographic planes of the PB tetragonal form II. In contrast, the diffraction peaks at 2θ = 9.9° of (110)_I/I′_ crystallographic planes in the PB trigonal phase were also observed in PB/PE(3/7), PB/PE(2/8), and PB/PE(1/9). The appearance of the trigonal phase is consistent with the additional melting peaks observed in the DSC heating curves (Figure 2a). It was indicated that the trigonal phase that was crystallized directly from the amorphous melt, referred to as form I′, is generated during the cooling crystallization of PB/PE(3/7) and PB/PE(2/8).

**Figure 4:**
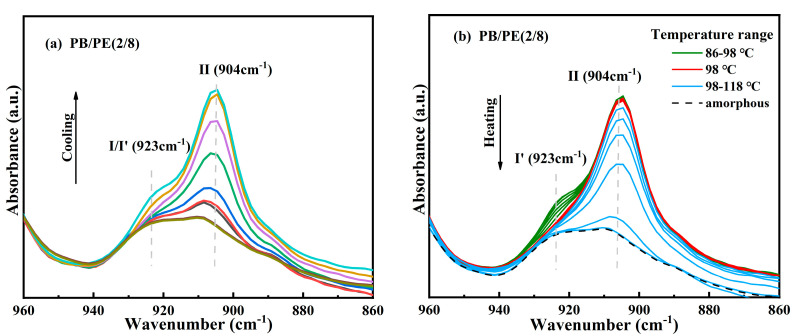
In situ FTIR spectra obtained within (**a**) cooling and (**b**) heating processes of PB/PE(2/8).

After that, to verify the modification of crystallites corresponding to the additional melting peaks at a low temperature, in situ FTIR experiments were carried out to closely track the real-time cooling crystallization and melting processes of PB/PE(2/8) and PB/PE(3/7). It can be seen from Figure 4a that, during cooling, the characteristic absorption band at the wavenumber of 904 cm^−1^, which originates from the tetragonal form II, increases earlier than that at 923 cm^−1^ of the trigonal phase. During the following heating (Figure 4b), the characteristic signal of the trigonal phase at 923 cm^−1^ began to decrease at 86 °C (green curves) and disappears completely at 98 °C (red curve). However, the characteristic signal at 904 cm^−1^ of form II remains unchanged during heating. The lower melting temperature indicates that all the trigonal crystals formed by cooling is form I′, whereas there is no form I transformed from form II. As the temperature was continuously elevated, the intensity of the form II absorption band at 904 cm^−1^ decayed and completely disappeared at around 118 °C, consistent with PB/PE(3/7) (Figure S1). These results demonstrate that the mixed crystals of forms II and I′ are generated for PE content ≥ 70 wt%, while only tetragonal form II can be formed during cooling crystallization in the blends with less than 70 wt% PE. This is different from the previous study where a presence of less than 30% PE can induce the appearance of PB form I′ in the cooling process [62]. In reality, it has been demonstrated by the systematic investigation on polybutene/propylene blends that the crystallization of polybutene is strongly dependent on the molecular weights of both PB and the matrix [55]. Thus, these different PE thresholds to induce form I′ might be associated with distinct molecular weights. In this work, the introduction of high PE content (≥70 wt%) greatly reduces the crystallization capacity of PB, as shown by the obviously decreased copolymer *T*_c_ with respect to pure PB (Figure 1b). In this case, the crystallization advantages of form II may reduce so much that form I′, which is the most thermodynamically stable, has the chance to grow detectably.

### 3.2. Polymorphism of Isothermal Crystallization

**Figure 5:**
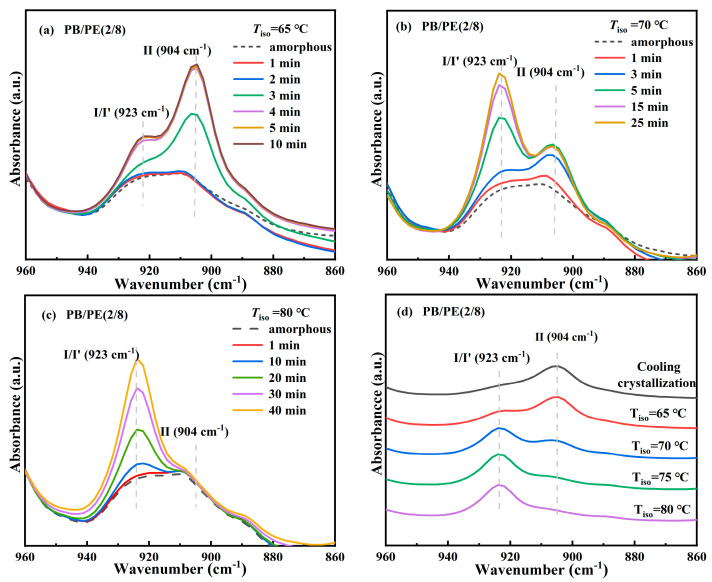
In situ FTIR spectra of PB/PE(2/8) obtained during isothermal crystallizations at (**a**) 65, (**b**) 70, and (**c**) 80 °C. (**d**) The summarized FITR spectra of PB/PE(2/8) after isothermal crystallization at different temperatures.

**Figure 6:**
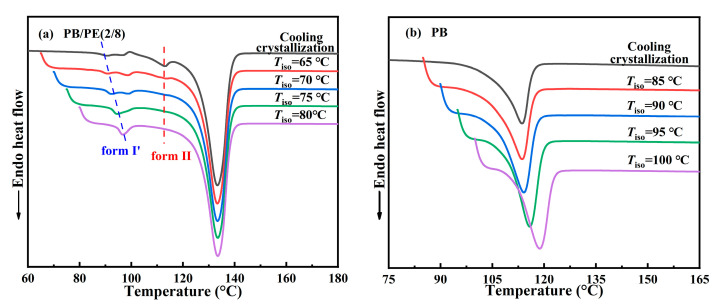
The DSC heating curves of (**a**) PB/PE(2/8) and (**b**) PB after isothermal crystallizations at different temperatures.

The above results clearly demonstrate the formation of form I′ in the blends with high PE fractions, including PB/PE(2/8) and PB/PE(3/7), so the isothermal protocol was further employed to reveal the correlation between polymorphism and temperature. The in situ FTIR characterization was employed to track the isothermal crystallization process, as shown by the representative of PB/PE(2/8). Figure 5a shows the in situ FTIR spectra acquired within the isothermal crystallization at 65 °C. It can be seen that the absorption band at 904 cm^−1^ and that at 923 cm^−1^ appear almost at the same time of 3 min. This means that in PB/PE(2/8), the trigonal phase appears simultaneous with form II within the isothermal crystallization at 65 °C. In this case, the origin of the trigonal phase, i.e., form I′ from melt crystallization or form I from II-I phase transition, cannot be distinguished. However, the relatively weak band of 923 cm^−1^, with respect to that of 904 cm^−1^, shows a small quantity of trigonal crystallites formed. As the isothermal temperature was elevated to 70 °C, a more trigonal phase was generated, which was indicated by the pronounced characteristic band at 923 cm^−1^ (Figure 5b). Based on the results of the corresponding heating (Figure S2), it is demonstrated that these trigonal crystallites with the lower melting temperature were form I′ that crystallized directly from the amorphous melt, rather than form I which was transformed from form II. At 75 °C, the more trigonal phase form I′ and the less trigonal phase form II were obtained (Figure S3). As the isothermal temperature was further increased to 80 °C (given in Figure 5c), only the absorption band of the trigonal phase was observed at 923 cm^−1^, which must be form I′. Thus, the correlation between polymorphism and temperature is summarized in Figure 5d. It is demonstrated that, for isothermal crystallization, the elevation of temperature was favorable for the formation of form I′ with respect to form II, and almost pure form I′ was generated for *T*_iso_ ≥ 80 °C. This correlation can also be confirmed by the DSC heating results. As shown in Figure 6a, the endothermic peaks within 86–98 °C of form I′ increases with elevating the isothermal temperatures, while those at 114 °C of form II show the opposite dependence. These results demonstrate that in the PB/PE(2/8) blend, the increase of the isothermal crystallization temperature is beneficial to the generation of form I′, in contrary to the case of pure PB with persistent form II (Figure 6b).

**Figure 7:**
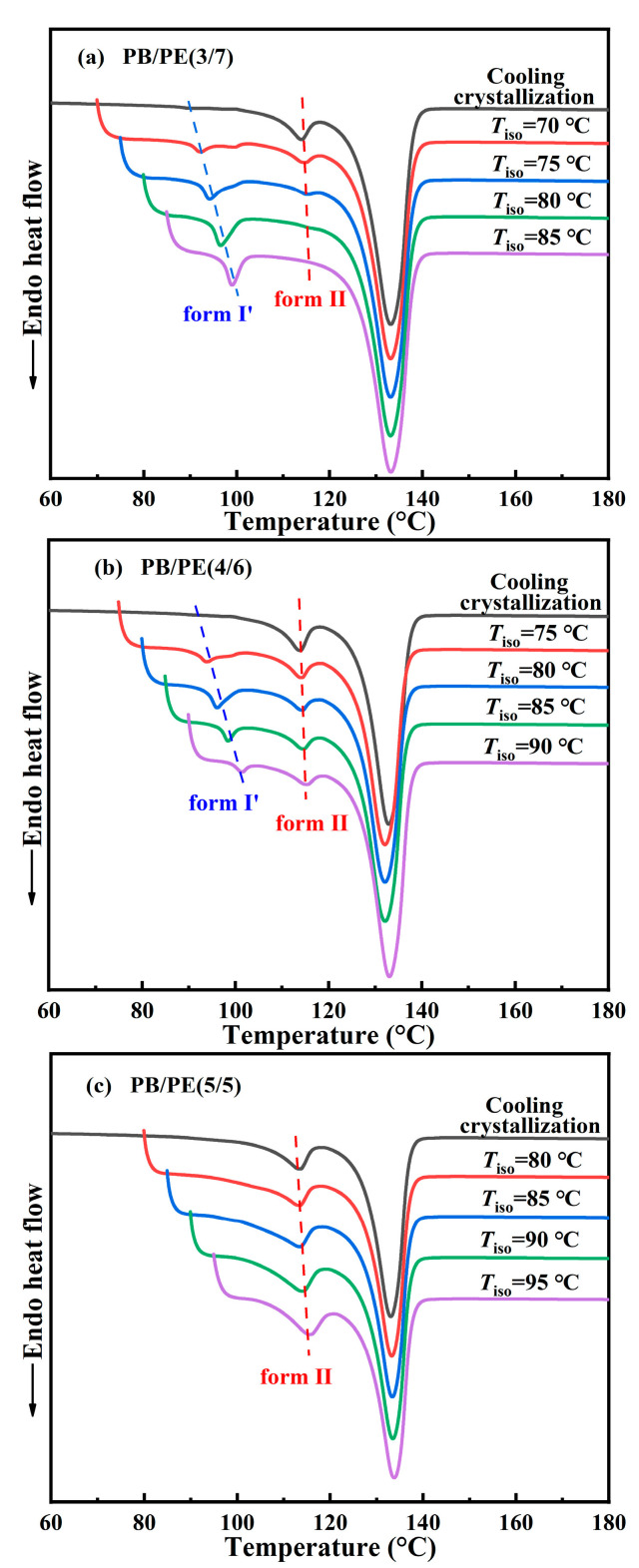
The DSC heating curves after isothermal crystallization at different temperatures for (**a**) PB/PE(3/7), (**b**) PB/PE(4/6), and (**c**) PB/PE(5/5).

Afterwards, the DSC experiments were utilized to examine the correlation between polymorphism and temperature for other blends. Figure 7a shows the DSC heating curves of PB/PE(3/7) obtained by isothermal crystallization at different temperatures. It can be seen that when the temperatures of isothermal crystallization are not higher than 80 °C, the DSC heating curves show both the melting peaks of form II and form I′, indicating that forms II and I′ are generated within the isothermal crystallization process. The content of form I′ increases with increasing isothermal temperature and pure form I′ can be obtained, similar to the above PB/PE(2/8). However, the lowest threshold temperature of obtaining pure form I′ increased to 85 °C for PB/PE(3/7) with respect to 80 °C for PB/PE(2/8).

Figure 7b presents the heating process of PB/PE(4/6) after the isothermal crystallization blend with the increased fraction of PB. It is interesting to observe that the isothermal crystallization at 75–90 °C largely promotes the formation of form I′, whereas the cooling crystallization generated pure form II (Figure 2a and Figure 3). Notably, in the PB/PE(4/6) blend, there are always the mixture of forms I′ and II for the approachable isothermal temperatures up to 90 °C. Furthermore, Figure 7c displays that in PB/PE(5/5), only form II was formed within the isothermal crystallization, similar to pure PB. The absence of form I′ was found in the isothermal experiments of blends with further reduced PE fractions below 50 wt% (DSC results of PB/PE(8/2) and PB/PE(7/3) are provided in Figure S4). 

### 3.3. Phase Transition from Tetragonal Phase into Trigonal Phase

**Figure 8:**
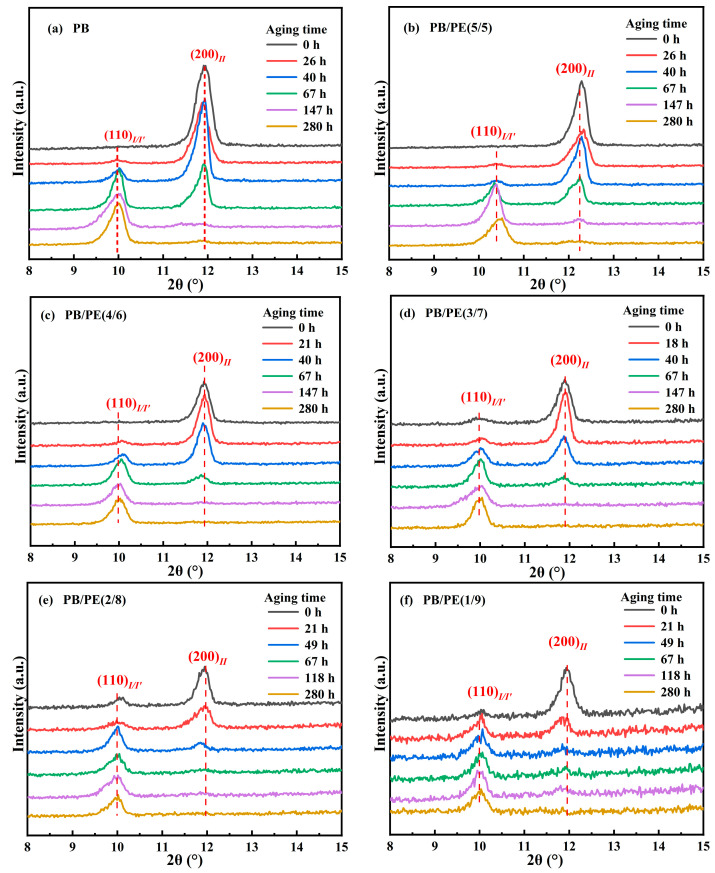
The XRD results of (**a**) PB, (**b**) PB/PE(5/5), (**c**) PB/PE(4/6), (**d**) PB/PE(3/7), (**e**) PB/PE(2/8), and (**f**) PB/PE(1/9) after aging at 25 °C for different durations.

It is known that the formed form II is not thermodynamically stable, and spontaneously transforms into form I. The solid phase transition was studied for form II crystallites obtained by cooling. Figure 8 shows the XRD diffraction of PB/PE blends after annealing at 25 °C for different durations. It can be seen that, after aging, the diffraction peak at 2θ = 11.9° of form II decreases but that at 2θ = 9.9° of form I/I′ increases. From Figure 8a,b and Figure S5, it seems that, for blends with no more than 50 wt% PE, their II-I transition kinetics are similar to pure PB. Interestingly, as a fraction of PE in the blend was increased to 60 wt% and more, the fraction of the transformed trigonal phase was higher than that of pure PB, indicating the accelerated transition kinetics.

**Figure 9:**
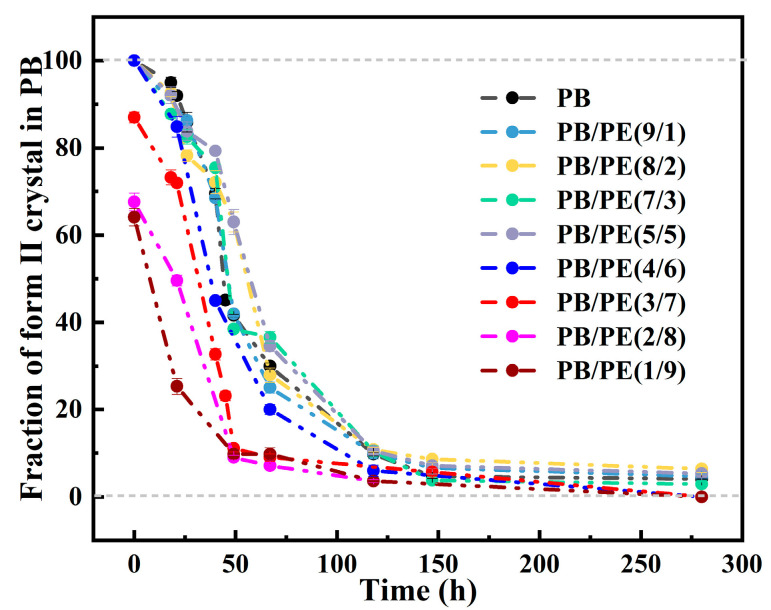
The evolution of form II fractions in total PB crystallites during aging at 25 °C.

To quantitatively compare the phase transition kinetics, the fractions of residual form II in crystal (*f*_II_) was determined from the XRD results. The equation employed was as follows [35]:

$$f_{\mathrm{II}}=(1-\frac{A{\left(110\right)}_{I/I^{\prime }}}{A{\left(110\right)}_{I/I^{\prime }}+0.67\times A{\left(200\right)}_{\mathrm{II}}})\times 100\%$$

where *A*(110)_I/I′_ and *A*(200)_II_ are the integral areas of the (110)_I/I′_ and (200)_II_ diffraction peaks, respectively. As shown in Figure 9, the transition kinetics are comparable for the PB/PE blends with no more than 50 wt% PE. When the PE content reaches 70 wt%, the phase transformation of form II into form I is obviously accelerated, with respect to pure PB, which further increases with adding more of the PE component.

## 4. Conclusions

A series of polybutene/polyethylene (PB/PE) blends with different proportions were prepared by solution blending. The crystallization behavior and II-I phase transition of PB blended with PE were studied by differential scanning calorimetry (DSC), in situ Fourier transform infrared spectroscope (FTIR) and X-ray diffraction (XRD). The results show that, in PB/PE blends, the cooling crystallization temperature of PB first increased and then decreased gradually with the increase in PE content, which had the highest crystallization temperature with 10 wt% PE. The XRD results show that, in addition to the kinetically favored form II, the trigonal phase also appears in the cooling crystallization of blends with 70–90 wt% PE. The in situ FTIR measurements were employed to track the heating process, and the results demonstrate that these trigonal crystallites all correspond to the form I′ that directly crystallized from the amorphous melt. Following this, the correlation between polymorphism and temperature was systematically studied with isothermal crystallization experiments. It was found that pure form II was generated with isothermal crystallization in the blend with ≤50 wt% PE, while form I′ was able to crystallize in the presence of 60–80 wt% PE. Interestingly, pure form I′ can be generated in blends with 70 and 80 wt% PE, of which the critical isothermal temperature should reach 85 and 80 °C, respectively. Furthermore, the influence of composition ratio on the solid phase transition from form II into form I was also investigated at 25 °C. It was found that, when PE content reaches 70 wt% and higher, the II-I phase transition kinetics of blends can be obviously accelerated with respect to PB.

## Data Availability

The data presented in this study are available on request from the corresponding author.

## Supplementary Materials

The following supporting information can be downloaded at: https://www.mdpi.com/article/10.3390/molecules27082448/s1, Figures S1–S3: FTIR results; Figure S4: DSC results; Figure S5: XRD results.

## Sample Availability

Samples of the compounds are available from the authors.
