# Supplementary material for: Crystallization Behavior of Isotactic Polybutene Blended with Polyethylene
Source: Molecules. 2022 Apr 11;27(8):2448. doi: 10.3390/molecules27082448 (PMC9028261; doi:10.3390/molecules27082448)
Supplement: Supplementary file 1 [file molecules-27-02448-s001.zip › molecules-1647196-supplementary.pdf]

Supporting Information

Crystallization behavior of polybutene blended with polyethylene

Jiajia Ping, Guiqiu Ma,* and Zhe Ma*

Tianjin Key Laboratory of Composite & Functional Materials, School of Materials Science and Engineering, Tianjin University, Tianjin 300350, China.

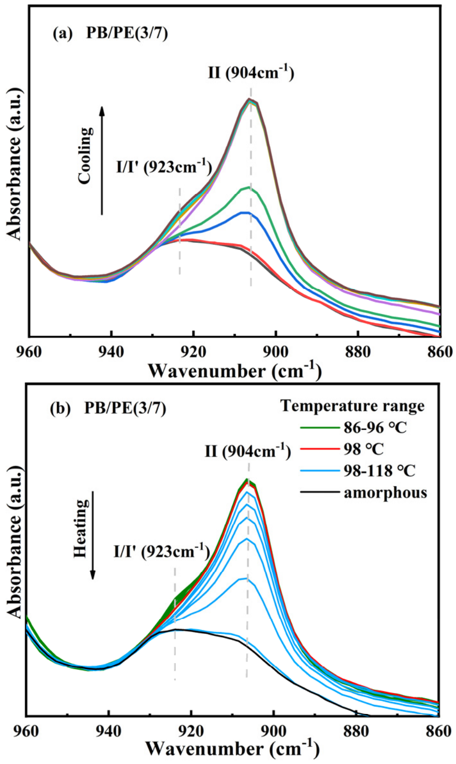

Figure S1. In-situ FTIR spectra of PB/PE(3/7) obtained during (a) cooling and (b) heating processes.

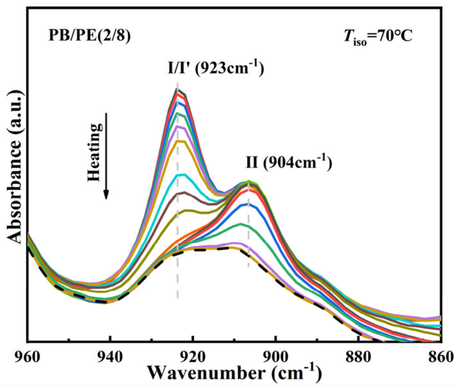

Figure S2. In-situ FTIR spectra of heating in PB/PE(2/8) after the isothermal crystallizations at 70 °C.

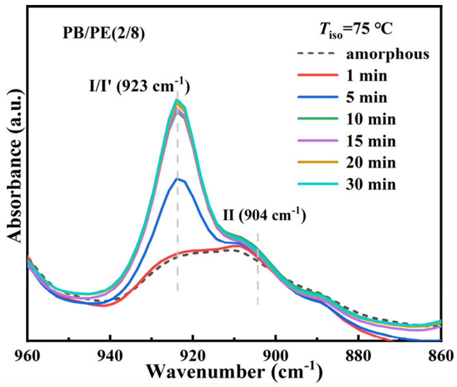

Figure S3. In-situ FTIR spectra of PB/PE(2/8) obtained during isothermal crystallizations at 75 °C.

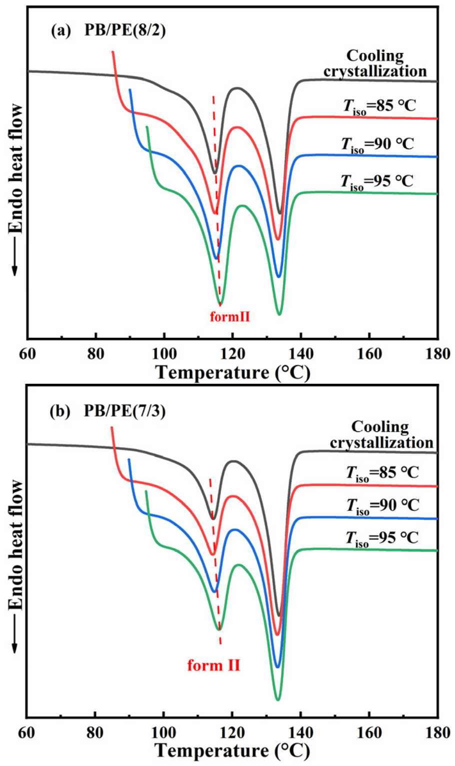

Figure S4. The DSC heating curves of (a) PB/PE(8/2) and (b) PB/PE(7/3) after isothermal crystallization.

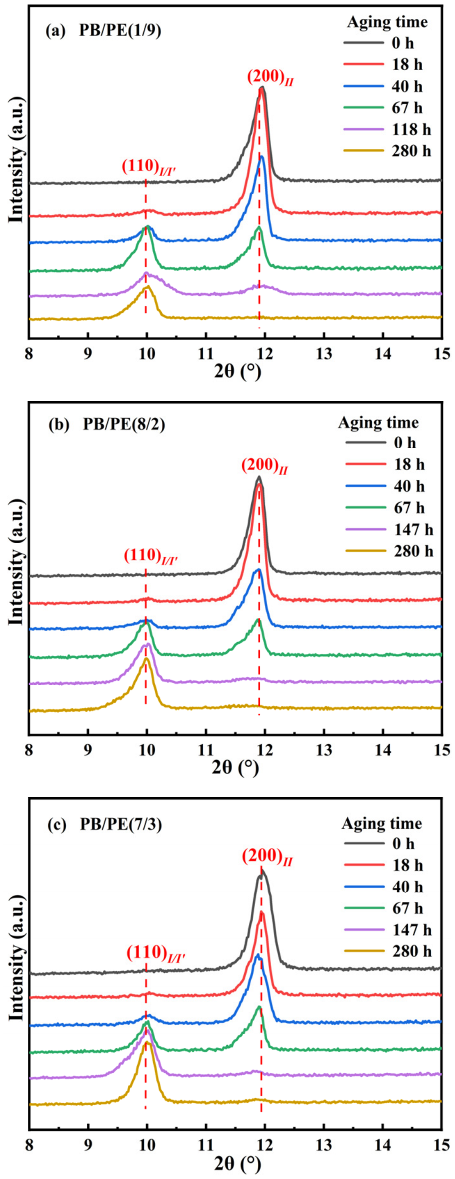

Figure S5. The XRD results of (a) PB/PE(9/1), (b) PB/PE(8/2), and (c) PB/PE(7/3) after aging at 25 °C for different durations.
